# Emotion recognition and human-robot interaction: a systematic review of methods and applications

**DOI:** 10.3389/frobt.2026.1891746

**Published:** 2026-09-11

**Authors:** Iraide Seijo Barquin, Juan Alejandro Castano, Virginia Ruiz Garate, Antonio J. del-Ama

**Affiliations:** 1 Robotics and Automation Department, Faculty of Engineering, Mondragon Unibertsitatea, Bilbao, Spain; 2 Applied Mathematics, Materials Science and Electronic Technology Department, Bioengineering Systems and Technologies Research Group (BeST), Rey Juan Carlos University, Móstoles, Spain; 3 Ikerbasque, Basque Foundation for Science, Bilbao, Spain

**Keywords:** affective computing, assistive robotics, emotion, facial recognition, multimodal interaction

## Abstract

Physical human–robot interaction involves a tightly coupled cognitive interaction with the user. The emotional state of the user is therefore a key factor influencing engagement, motivation, and long-term acceptance. This review explores recent developments in emotion-aware technologies, particularly facial expression recognition and multimodal affect detection, and their potential to make assistive robots more adaptive and human-centred. By analysing current methodologies, emerging trends, and ethical considerations, we show how the integration of emotional intelligence can transform robotic assistance, shifting from reactive mechanisms to genuinely empathetic, socio-psychological, and context-aware assistive partners.

## Highlights

- This review provides a comprehensive categorization of physiological and behavioral signals specifically tailored for Human-Robot Interaction (HRI) and assistive contexts.
- It identifies critical technological gaps, such as the current lack of closed-loop affective control, that hinder the transition of robotic systems from laboratory settings to real-world clinical and social environments.
- The work offers a strategic roadmap for integrating emotional intelligence into assistive robotic architectures, prioritizing user safety, trust, and long-term socio-psychological acceptance.

## 1 Introduction

Human physical performance is mainly influenced by physical state. Yet, emotional factors such as motivation, frustration, and confidence have been shown to significantly affect motor behavior (Cappellini et al., 2006), learning capabilities, and engagement with technology (Marina-Miranda and Traver, 2022; Chen et al., 2024b). Recent research in human–robot interaction (HRI) and motor learning further supports this view. Specifically, emotional and motivational factors such as frustration, perceived challenge, and sense of control have been found to substantially shape motor performance and thus the effectiveness of robotic assistance (Hwang et al., 2022; Wu, 2021). Individual differences in personality traits, including locus of control and exploratory tendencies, also influence how users interact with assistive robots, how much physical effort they exert, and how efficiently they learn. In areas such as rehabilitation, users experiencing high frustration or low perceived control often display reduced engagement and poorer retention. Conversely, appropriate modulation of task difficulty or robotic assistance can restore motivation by rebalancing the challenge-skilled ratio (Garzás-Villar et al., 2025). These findings indicate that emotional responses are not mere by-products of interaction but active determinants of behavioral adaptation. This, underscores the need for human-state-aware robotic controllers capable of supporting optimal engagement and learning conditions. In collaborative scenarios such as robot assisted rehabilitation, the inability of a robot to sense user frustration can lead to misaligned assistance, potentially compromising both rehabilitation outcomes and physical safety.

Given the variety of terminology used in this field, the following terms are used consistently throughout this review: Affective computing refers to the research and development of systems that address human feelings, emotions, attachments, or moods; Emotion(al) recognition/estimation refers to the various technical methodologies employed to detect these states, whereas, emotional intelligence refers to the ability of a person or system to behave appropriately in response to perceived emotions. Where specific literature is discussed, however, the terminology used in the corresponding work has been preserved.

The influence of emotional states on human performance can be better understood by considering psychological theories of emotion, which describe emotional experience as emerging from the interplay of physiological responses, perception, and cognitive appraisal. The James–Lange theory proposes that emotions arise from the perception of physiological changes (Hiatt et al., 2011), whereas Cannon–Bard’s argues for the simultaneous yet independent processing of cognitive and emotional responses (Bratman, 1987). These perspectives clarify why emotions are consistently expressed through identifiable behavioral, physiological, and expressive patterns. Since such patterns correspond to measurable bodily and behavioral signals, they offer a conceptual foundation for computational approaches to emotion detection. Empirical findings further support these relationships. Fostering specific emotional states, such as hope, can positively influence functional outcomes in rehabilitation (Kortte et al., 2012). Moveover, human-state-aware robots have demonstrated the ability to adapt their behavior based on psychophysiological indicators, including heart rate and skin conductance (Guerrero et al., 2013).

The integration of emotional information into interactive technologies has advanced considerably over the past decade, driven largely by progress in automatic emotion recognition (AER), including facial emotion recognition (FER). Modern AER/FER systems analyze signals from computer vision, speech, and physiological sensing to characterize both discrete emotions (e.g., happiness and frustration) and continuous affective dimensions (e.g., valence and arousal. Early approaches relied mainly on static facial images, which limit their application in dynamic or complex contexts (Luna-Jiménez et al., 2021; Su et al., 2021). Recent developments incorporate temporal information and multimodal cues, giving rise to more robust and context-aware analysis. Deep Learning (DL) architectures, including Convolutional Neural Networks (CNNs), Recurrent Neural Networks (RNNs), and transformer-based models, have significantly improved the representation and classification of emotional information (Othmani et al., 2021; Smith et al., 2020). These advancements have facilitated a shift toward systems able to handle occlusion and lighting variations typical of real-world assistive environments, where a robot must maintain emotional tracking while moving or interacting.

As these methods have evolved, technolgoies capable of integrating multimodal data have emerged. For instance, interactive systems such as social and assistive robots, adaptive educational platforms, and affect-sensitive human–computer interfaces can leverage multimodal emotional information to adjust behavior, feedback, or level of assistance in real time. These systems combine physical indicators, such as posture, muscle activation, eye movement, skin temperature, and gait patterns, with expressive and emotional cues, including facial expressions, tone of voice, and touch-based interactions (Heredia et al., 2022; Bokhare and Kothari, 2023; Deepika and Kuchibhotla, 2023). This combination provides richer, more context-aware, and personalized responses. Multimodal fusion through DL further enhances robustness, real-time adaptability, and user satisfaction. However, these advances also introduce challenges, such as handling missing or low-quality sensor inputs (Kahou et al., 2013), adapting solutions to diverse demographic and contexts. Emotional detection methods must also be validated longitudinally to assess reliability over time.

Emotions therefore play a crucial role in technology acceptance, as user experience remains the key consideration for human–technology interaction. Although multimodal systems aim for technical robustness, studies show that their success often depends on overcoming persistent issues such as low user satisfaction and the lack of intuitive, user-centered evaluation tools (Schneider et al., 2016; Lohse et al., 2014; Herrera-Valenzuela et al., 2023). By integrating emotional awareness, these systems can better align with real-world user needs. This alignment is essential for moving beyond simple usability toward long-term acceptance and perceived usefulness (Schneider et al., 2016; Herrera-Valenzuela et al., 2023).

Hence, integrating emotion-aware systems into physically and socially interactive robots represents a promising approach to enhancing personalization, adaptability, and meaningful user engagement across application domains (Dikbiyik et al., 2025; Subramanian et al., 2022). A recent systematic review identified specific visual factors that trigger positive emotional responses in immersive environments, providing a foundation for the design of more effective affective interfaces (Goates et al., 2026). Evidence from assistive and therapeutic robotics indicates that adapting to the affective state of the user directly influences interaction outcomes. Reported benefits include increasing patient motivation during rehabilitation (Tapus et al., 2007), enhancing engagement in clinical interventions (Scassellati et al., 2012), andreduced user frustration (Feil-Seifer and Mataric, 2005). Taken together, this foundational literature, which falls outside the scope of the present systematic corpus, suggests that emotion-aware behavior should be a core feature of assistive and rehabilitation robotics. As detailed in Section 3.2.3, however, our systematic review finds that this connection remains empirically thin within the emotion-recognition literature itself: closed-loop integration between recognized affect and rehabilitation-relevant robotic action is, to date, largely undemonstrated.

Despite the demonstrated benefits of emotional state integration in HRI, most existing surveys focus heavily on algorithmic accuracy. Accordingly, this review provides a comprehensive overview of current online emotional estimation methods essential for real-time integration into robotic control loops. It examines their application fields, performance, and associated challenges, with particular emphasis on their relevance to human–technology interaction. In contrast to traditional surveys, we adopt an application-first approach that focuses specifically on the deployment of emotion recognition within the control loops of assistive robotics. We analyze how these methods transition from laboratory-based experiments to real-world socio-psychological support in healthcare and HRI. Furthermore, we provide a curated selection of emotion databases to support the development of more context-aware robotic controllers and assist, researchers and engineers seeking to validate affective models in complex, human-centered environments. It should be noted that a substantial share of the reported literature originates from general Human-Computer Interaction or emotion recognition methods and benchmarking studies, not yet deployed on physical robotic platforms. These have been included due to their potential for direct transfer to HRI and assistive robotics applications.

## 2 Methodology

This review consisted of two sequential phases. The first exploratory phase mapped the conceptual landscape of the field, examining how research linking human interaction with technology has addressed emotional dimensions, particularly those associated with mental health and performance outcomes. The second phase utilized the refined and more focused set of terms derived from the first phase to perform the formal search and screening process of the review.

### 2.1 Type of review

This work is conducted as a systematic literature review, following the PRISMA (Preferred Reporting Items for Systematic Reviews and Meta-Analyses) guidelines (Moher et al., 2009). Unlike a scoping review, which aims to map the breadth of a research area without necessarily assessing methodological quality (Arksey and O’Malley, 2005), or a systematic mapping study, which classifies the structure and trends of a research field at a coarser granularity (Petersen et al., 2008), a systematic review applies pre-specified eligibility criteria and a structured screening protocol to synthesize the state of the art on a well-defined research question (Kitchenham and Charters, 2007). This distinction motivated the two-phase design adopted here: an initial exploratory mapping phase, used only to delineate the scope and terminology of the field, followed by a formal systematic review phase, which applied the full PRISMA protocol and eligibility criteria to produce the final set of 60 included studies.

### 2.2 Exploratory mapping phase

During the first phase, an exploratory search was conducted across Scopus (October 2024), IEEE Xplore, and PubMed to map the landscape of emotion recognition and human–technology interaction. This initial stage relied on inclusive, loosely defined keywords combining terms such as cognitive performance and mental health with technical descriptors like facial action coding and 3D facial analysis. No inclusion/exclusion filtering was applied at this stage beyond basic relevance screening by title. This process led to the identification of 39 representative records (summarized in Table 1). These records were then reviewed qualitatively to extract recurring application domains and methodological trends, providing the thematic foundation for the more restrictive search strategy that followed.

**TABLE 1:** Inclusion and exclusion criteria applied during the systematic search and screening process.

Stage	Criterion	Type
Identification	Publication date range: 2020–2025	Automatic (database/Zotero filter)
	Document type: journal articles, conference proceedings, edited books, magazines	Automatic (database/Zotero filter)
	Duplicate removal across databases	Automatic (zotero)
Screening	Written in English	Manual (reviewer judgement)
	Openly accessible	Manual (reviewer judgement)
	Excludes reviews, meta-analyses, and surveys	Manual (reviewer judgement)
	Thematic relevance to emotion/facial expression recognition within a human–technology interaction context	Manual (independent dual screening)
Included	Original empirical work only (excludes purely theoretical/conceptual papers)	Manual (independent dual screening)
	Uncertain cases cross-validated by a second reviewer	Manual (dual reviewer consensus)

The studies identified during this exploratory phase were not directly included in the formal screening or quantitative analysis. They were used solely to refine the scope of the review and to calibrate the final Boolean search toward concepts directly related to human–technology interaction and facial expression analysis.

### 2.3 Eligibility criteria

**FIGURE 1:**
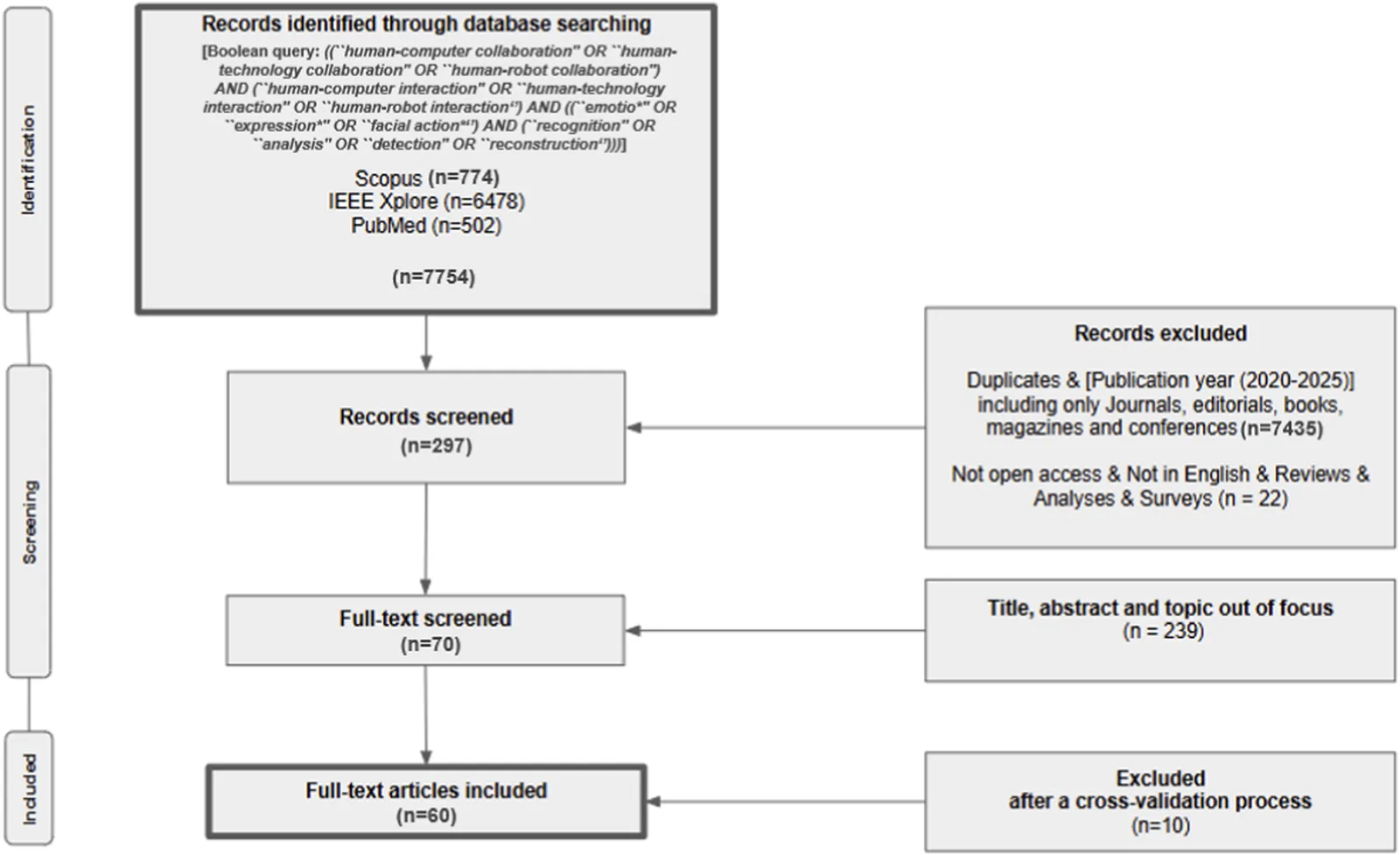
PRISMA flow diagram illustrating the consulted databases, the reviewed bibliography, and the study selection process. This diagram followed the guidelines for reporting systematic reviews of (Page et al., 2021).

The exploratory search refined our strategy, narrowing the focus to computational and interactional aspects of emotional recognition across diverse technologies. With this refined scope, the second phase was carried out, with a comprehensive and systematic search following the PRISMA guidelines (see Figure 1).

The systematic search was conducted between November 2024 and August 2025 in Scopus, IEEE Xplore, and PubMed. The search was conducted using the following Boolean query: ((“human-computer collaboration” OR “human-technology collaboration” OR “human-robot collaboration” OR “human-computer interaction” OR “human-technology interaction” OR “human-robot interaction”) AND ((“emotio*” OR “expression*” OR “facial action*“) AND (“recognition” OR “analysis” OR “detection” OR “reconstruction”))).

Studies were eligible for inclusion if they were published between 2020 and 2025 in peer-reviewed journals, conference proceedings, or edited books; were written in English; were openly accessible; and reported original empirical work (excluding reviews, meta-analyses, and surveys) addressing emotion or facial expression recognition within a human–technology interaction context (human–computer, human–robot, or human–technology interaction broadly). Studies were excluded if their title, abstract, or full text indicated a primary focus outside this scope, or if, after full-text review, their thematic relevance to the review’s focus could not be confirmed.

The first search identified a total of 7,754 records: 502 from PubMed, 774 from Scopus and 6,478 from IEEE Xplore. After removing duplicates across databases and restricting the publication date range to 2020–2025 (including only journals, editorials, books, magazines, and conference proceedings), 323 records remained. A further 22 records were excluded for not being openly accessible, not being written in English, or corresponding to reviews, meta-analyses, or surveys, resulting in 297 records for title/abstract screening.

Table 2 summarizes the full set of inclusion/exclusion criteria applied throughout the search and screening process, distinguishing automatic (database-level) filters from manual, reviewer-applied criteria. In particular, purely theoretical or conceptual contributions (i.e., studies not reporting original empirical work, such as proposals, position papers, or opinion pieces without an implemented and evaluated system) were excluded via the eligibility criterion requiring original empirical work, applied during full-text screening.

**TABLE 2:** ROBIS assessment of the present systematic review.

ROBIS domain	Judgement	Rationale
1. Study eligibility criteria	Low concern	Eligibility criteria (publication years 2020–2025, peer-reviewed, English, open access, original empirical work) were pre-specified and reported in the eligibility criteria subsection prior to screening
2. Identification and selection of studies	Low concern	A comprehensive boolean search was run across three databases (scopus, IEEE xplore, PubMed); duplicate removal and screening were performed independently by two reviewers using zotero, with discrepancies resolved by discussion
3. Data collection and study appraisal	Mid concern	Data items were extracted independently by two reviewers for all included studies; however, a formal study-level quality appraisal was introduced during the revision process rather than during the original extraction phase, and is reported *post hoc* in Supplementary Appendix A
4. Synthesis and findings	Low concern	Given the methodological heterogeneity of included studies, a narrative/thematic synthesis, grouped by application domain, was used instead of quantitative pooling, which is appropriate for this type of heterogeneous corpus

### 2.4 Selection and data extraction process

Reference management and duplicate removal were performed using Zotero (Corporation for Digital Scholarship). Title/abstract and full-text screening were independently conducted by two of the authors. Discrepancies regarding inclusion or exclusion were resolved through discussion, prioritizing alignment with the thematic scope defined in the exploratory phase. Boolean query variations were also tested iteratively during this process, as alternative keyword combinations were found to omit relevant studies from adjacent research areas.

These 297 records were screened by title, abstract, and topic focus (medicine or engineering), resulting in the exclusion of 239 records whose title, abstract, or topic fell outside the scope of the review. A total of 69 articles remained for full-text assessment. Subsequently, 10 uncertain papers were cross-validated by a second reviewer and were discarded as being out of scope. Thus, 60 articles were ultimately included in the final review.

For the 60 included studies, both reviewers independently extracted the following data items: application domain, input modality, specific application/use case, algorithmic approach, dataset(s) used, presence and characteristics of human participants/subjects, and reported system accuracy. These data are summarized in Tables 1, 3 in the Results section: Table 3 reports the results of the final PRISMA-based search described above, while Table 1 reports the earlier, more exploratory search that preceded the definitive Boolean query. This exploratory search was not used for the final quantitative screening but is reported for completeness and transparency.

**TABLE 3:** First research results: Compact emotion recognition Literature Review.

File	Input	Application	Algorithmic choice	Database	Subjects	Max. Accuracy
Mehendale (2020)	FER	No App	CNN (two-stage)	(1)/(2)/(3)/(4)	H	96%
Yang et al. (2020)	FER	HCI	Exchange-GAN	(3)/(42)/(86)	H	95.24%
Nayak et al. (2021)	FER	HCI	Faster R-CNN + DTW	(43)/Own	H	-
Modi and Bohara, (2021)	FER	No App	CNN	(10)	H	0.82521
Liu et al. (2021)	FER	Education	CDLLNet (GoogleNet + Cauchy labels)	(86)/(1)	H	87.42%
Hung and Chang, (2021)	FER + NER	No App	mLTL	(1)/(10)	H	87.84%
Sun et al. (2021)	FER	HCI	MSDModel	(1)/(23) (86)	H	99.10%
Jin and Jin, (2021)	FER	HCI	MiniExpNet (60K params) + HLBP	(1)/(29)/(86)	H	96.18%
Li et al. (2021)	FER	No App	LBAN-IL	(24)/(26)/(10)/(30)	H	85.89%
Lakshmi and Mohanaiah, (2021)	FER	HCI	WOA-TLBO + Multi-SVNN	(1)/(27)/(28)	H	90%–98%
Sharafi et al. (2022)	Multimodal	HCI	CNN + BiLSTM fusion	(11)/(25)/(47)	H	99.75%
Magherini et al. (2022)	FER	HCI	ResNet50v2 + InceptionResNetV2	(13)	H	96.9%
Gan et al. (2022)	FER	HCI	DenseNet + hierarchical attention	(1)/(24)	H	95.71%
Ullah et al. (2022)	FER	HCI	DCNN + RNN + BiGRU (score fusion)	(27)/(1)	H	95%–96%
Torre-Luque et al. (2022)	FER	Healthcare	FERT + Questionnaires	(108)	P	-
Zhang and Yu (2022)	FER	HCI	EFE + EMR + EPMG	(1)/(86)/(24)/(13)	H	98.06%
Huang et al. (2023)	FER	Healthcare	Haar Cascade + MobileNetV2	Own	P	80%–82%
Bisogni et al. (2023)	FER	Surveillance	Landmarks + CNN + LSTM	(1)	H	48.4%
Luzio et al. (2023)	FER	Healthcare	Randomized DNN +468 landmarks	(1)	H	(0.889–0.986)
Hossain et al. (2023)	FER	No App	CNN + CNNBilinear	(37)/(26)/(31)	H	80.34%
Chen et al. (2024a)	FER	HCI	GCN + dual subspace manifold	(1)/(86)/(23)/(90)	H	98.37%
Gong et al. (2024)	FER	Healthcare	ESTLNet (transformer)	(81)/(7)/(1)/(86)	H	99.04%
Tao and Duan, (2024)	FER	HCI	Hierarchical attention + progressive fusion	(1)/(13)/(10)/(26)/(24)	H	92%
Kırbız, (2025)	FER	HCI	CNN + RetinaFace	(1)/(10)/(6)	H	82%
Kim and Choi, (2025)	FER	HCI	TriBAN + GCN	(24)/(13)/(10)	H	91.43%
Mao et al. (2025)	FER	Industry	POSTER++ (MSI + window cross-attn)	(24)/(13)/(46)	H	93.00%
Wei et al. (2024)	FER (facial + EEG)	HCI	SISTCM + two-stream LSTM + ACCM	(1)/(91) - (95)	H	97.79%
Garg and Verma, (2020)	ER (EEG)	AC	Wavelet + CNN (GoogleNet)	(38)	H	92.19%
Tan et al. (2021)	ER (EEG)	HCI	Spiking NNs (eSNN, deSNN, NeuCube)	(38)/(39)	H	79.39%
Miao et al. (2023)	FER (EEG)	HCI	3D residual CNN	(40)/(41)	H	96.7%
Mutawa and Hassouneh, (2024)	ER (EEG)	Healthcare	Landmarks + EEG + Log-sync + LSTM	Own	H	99.3%
Liu et al. (2024)	ER (EEG)	HCI	MAS-DGAT-Net	(40)/(41)/(38)	H	99.86%
Lian et al. (2024)	Multimodal	No App	GPT-4V multimodal	(1)/(13/)/(24) - (26)/(81) - (83)/(95)- (107)	H	97.81%
Meléndez et al. (2020)	Emotion perception	Healthcare	Behavioral task	(89)	H	-
Nag et al. (2020)	ER + gaze tracking	Healthcare	Eye-tracking + CV	(34)	P	71%
Heredia et al. (2022)	Multimodal	Social	EmbraceNet + fusion	(9)	H	78.9%
Subramanian et al. (2022)	ER	Healthcare	MediaPipe + DL	Own	H	99%
Fu et al. (2023)	Mimicry	Healthcare	Human evaluation	Own	P	-
Gan et al. (2023)	FER	HCI	AR	-	H	-

CV, Computer Vision; DTW, Dynamic Time Warping; R-CNN, Regions with CNN features; Faster R-CNN, Faster Regions with CNN; GoogLeNet, Google Inception CNN; NER, Named Entity Recognition; LSTM, Long Short-Term Memory network; MSDM, Multi-Scale Depth Map; HLBP, High-dimensional Local Binary Patterns; Multi-SVNN, Multi-channel Support Vector Neural Network; InceptionResNet, Inception Residual Network; VGGFace, Visual Geometry Group Face Network; DCNN, Deep CNN; SVM, Support Vector Machine; MobileNetV2, Mobile Convolutional Neural Network version 2; DNN, Deep Neural Network; MLP, Multi-Layer Perceptron; DCGAN, Deep Convolutional Generative Adversarial Network; TGCN, Temporal Graph Convolutional Network; ACCM, Attention-Constrained Convolutional Module; POSIT, Pose from Orthography and Scaling with Iterations; MSI, Multistream Information; AC, Affective Computing; LBAN-IL, Local Binary Attention Network with Islets Loss; WOA-TLBO, Whale Optimization Algorithm with Teaching-Learning-Based Optimization; POSTER(+), Pyramid crOss-fuSion TransformER network; MAS-DGAT-Net, Multibranch And Staged fusion - Dynamic Graph ATtention Network; ResNet50v2, Residual Network 50-layer, version 2; DenseNet, Densely Connected Convolutional Network; BiGRU, Bidirectional Gated Recurrent Unit; GCN, Graph Convolutional Network; Haar Cascade, Haar feature-based Cascade classifier; SNN, Spiking Neural Network; eSNN, evolving Spiking Neural Network; deSNN, dynamic evolving Spiking Neural Network; NeuCube, Brain-inspired Spiking Neural Network architecture (proper name); GPT-4V, Generative Pre-trained Transformer 4 with Vision; EmbraceNet, Multimodal fusion neural network (proper name); MediaPipe, Google’s cross-platform ML pipeline framework (proper name); AR, Augmented Reality. Subjects Column: H: Healthy People / P: Patients

### 2.5 Synthesis methods

Given the methodological heterogeneity of the included studies, spanning multiple technologies, algorithmic approaches, and application domains, a narrative and thematic synthesis was performed rather than a quantitative meta-analysis. Studies were grouped by application domain and compared qualitatively in terms of algorithmic approach, dataset characteristics, and reported performance metrics.

### 2.6 Risk of bias and reporting bias assessment

The risk of bias of this systematic review was appraised using the four domains of the ROBIS tool (Risk of Bias in Systematic Reviews) (Whiting et al., 2016): (1) study eligibility criteria, (2) identification and selection of studies, (3) data collection and study appraisal, and (4) synthesis and findings. The corresponding judgement for each domain, together with the supporting rationale, is reported in Table 4.

**TABLE 4:** Compact emotion recognition Literature Review.

File	Input	Application	Algorithmic choice	Database	Subjects	Max. Accuracy
Nawaz et al. (2025)	FER	HCI	MTCNN	(5)/(1)/(13)/(7)/(24)	N	99.52%
Kansizoglou et al. (2022)	FER	HRI	Viola–Jones Cascade classifier	(16)	H	(0.676)
Dar et al. (2022)	FER	No App	EfficientNet + SwishNet	(27)/(9)/(6)/(10)/(54)	N	97.16%
Pham et al. (2019)	FER	No App	CNN + MLP	(10)/(15)	N	73.02%
Borgalli and Surve, (2022)	FER	No App	Standard CNN	(1)/(23)/(55)/(56)	N	74%
Kapaliya et al. (2024)	FER	HCI	VGG16	(10)/(5)	N	89%
Saurav et al. (2021)	FER	No App	DLTP + PCA + K-ELM	(1)/(57)/(6)/(27)/(24)	N	90.28%
Su et al. (2021)	FER	HCI	RHPN	(86)/(24)	N	99.75%
Alharbawee and Pugeault, (2024)	FER	No App	DCGAN + SVM	(29)/(60)/(24)/(6)/(37)/(1)	N	99.8%
Avani et al. (2021)	FER	HCI	Parabolic model + SVM/MLP/RF	(1)/(27)	N	96%
AlEisa et al. (2023)	FER	HCI	HGSO-DLFER	(1)	H	98.65%
Hwang et al. (2022)	ER (facial + speech)	HRI	SRCN-DFER + SRCN-WSCR	(59)/((6))/(27)/(1)/(88)	N	98%
Alrowais et al. (2023)	FER	HRI	MEOWDL-ER	(1)	N	98.91%
Dong et al. (2023)	FER	HCI	Multi-Net fusion	(10)/(1)/(27)	N	99.24%
Vo et al. (2020)	FER	HCI	PSR	(24)/(10)/(13)	N	89.75%
Luna-Jiménez et al. (2021)	Multimodal	HCI	Transformer Wav2Vec2.0	(25)	N	86.7%
Marinova et al. (2025)	FER	Wearable	CNN, ConvLSTM, STResNet	Own	H	HAR F1 = 0.88
Subramanian et al. (2022)	FER	Healthcare	CNN	Own	H	-
Morales-Vargas et al. (2019)	FER	No App	Blurred explanatory models	(1)/(29)	H	90.8%–92.9%
Li D et al. (2022)	ER (EEG + facial)	Rehab	CBAM_ResNet34 + fusion	Own	P	78.32%
Colaco and Han, (2025)	FER	HCI	Mini-Xception + modified EfficientNet	(78)	H	83.7%
Behzad, (2025)	FER	No App	SMILE-VLM	((68))/((70))/((69))/((51))	N	96.57%
Li X et al. (2022)	FER	No App	Res3D+SCA	(51)	H	85.59%
Choi and Song, (2020)	FME	HCI	CNN + LSTM	(45)/(82)/(83)/(1)/(39)/(79)	N	93%
Kim et al. (2021)	VAR + Postural	No App	ResNext-101	(66)	N	95.83%
Kang and Kim, (2022)	ER (biosensors)	HRI	1D convolutional autoencoder + CNN	(87)/(38)	H	79%–81%
Yildirim et al. (2021)	ER (EEG)	HCI	Swarm intelligence + kNN	(38)	H	79.07%
Gilakjani and Al Osman, (2023)	ER (EEG)	HCI	GNN	(38)/(39)	H	64%–80%
Li et al. (2024)	ER (speech)	HCI	CNN (A_Inception + MCA/TCA + center loss)	(9)/(35)/(11)	N	73%–92%
Cao et al. (2025)	ER (speech)	HCI	HuBERT + swin transformer + fusion HSF	(25)/(35)/(11)	N	79%–97%
Guo et al. (2023)	ER (speech)	HCI	Enhanced ResNet + multiband fusion	(35)/(25)/(9)	N	-
Ding et al. (2024)	Multimodal	Safety	AM-DSCNN + SVM + RF + RL	(13)/(72)/Own	H	91.68%
Marimpis et al. (2020)	ER (EEG)	No App	DoCM + FI/DFA + ELM-RBF	(38)	H	MSE: 0.56–1.96
Er, (2020)	ER (speech)	HCI	CNN + acoustic features + SVM	(25)/(35)/(9)	N	79%–90%
Ghous et al. (2025)	ER (EEG)	No App	Transformer with attention	(41)/(73)/(85)	H	79%–90%
Dikbiyik et al. (2025)	ER (speech + text)	HCI	ResNet50-CRNN + BERT + fusion	(9)	N	88.33%
Sajjad and Kwon, 2020	ER (speech)	HCI	CNN + BiLSTM + clustering RBF	(25)/(35)/(9)	N	72%–85%
Bagherzadeh et al. (2024)	ER (EEG)	HCI	CNN + LSTM + ensemble	(38)/(39)	H	98.8%
Mai et al. (2025)	ER (EEG)	HCI + AC	1D-CNN + self-knowledge distillation	(38)/(40)/(84)	H	98%–99%
Thant and Panitanarak, (2025)	ER (EEG + ocular)	No App	Kolmogorov-arnold networks	(73)	H	96%
Deeb et al. (2025)	ER (speech)	HCI	SSL + cross-attention	(9)	N	74.6%
Priyadarshani et al. (2024)	ER (EEG)	Healthcare	PCA + LSTM/GRU	Own	H	96%–97%
Chen et al. (2024b)	ER	HCI	End-to-end transformer	Text datasets (3)	N	89.9%–91.4%
Ho et al. (2020)	ER (speech + text)	HCI	RNN + multi-head fusion	(9)/(18)/(61)	N	65.9%
Shahzad et al. (2023)	ER (audio + facial)	HCI	CNN mod. Xception	(80)/(62)	H	81%–86%
Wang et al. (2023)	ER (EEG + facial)	HCI	CNN + attention + merger	(38)/(39)	H	79.81%
Wu et al. (2020)	ER (EEG + facial)	HCI	Hierarchical LSTM + attention	Own	H	96%–97%
Dal Rí et al. (2023)	ER (speech)	HCI	CNN + attention block (CBAM)	(25)/(62)/(9)	N	63%–100% **
Li et al. (2025)	Multimodal	Healthcare	MDT-GCN	(75)/(68)	N	90.11%
Deng et al. (2019)	FER	HRI	cGAN	(24)/(13)	N	81.83%
Pu and Nie, (2023)	FER	HCI	Xception + ResNet + SENet	(10)/(1)/(24)	N	99.61%
Sohail et al. (2024)	FER (postural + facial)	Biometric	Modified YOLO-V5	Own/(52)	N	98.5%
Lai et al. (2020)	Multimodal	HCI	DCWS-RNNs	(9)/(53)	N	64.45%
Marina-Miranda and Traver, (2022)	FER	HRI	CAUDHE + LSTM	Own	N	90%
Goncalves et al. (2025)	Multimodal	HCI	Multimodal transformers	(62)	N	84.5%
Sun and Cai, (2024)	FER (facial + ocular)	HCI	VGG-19	Own	N	99.04%
Jaffar et al. (2024)	Multimodal	HRI	Multimodal system (voice/face/text + ML)	Own	H	-
Radoi and Cioroiu, (2024)	Multimodal	HCI	Lightweight multimodal neural network	(61)/(25)	N	84.3%–86.7%
Güler and Akbulut, (2025)	Multimodal	HCI	GRU + LSTM + transformer	(38)/Facial videos	N	97.8%
Handrich et al. (2021)	FER	HRC	Tiny YOLOv3	(7)/(13)/(92)	Y	79%

MTCNN, Multi-task Cascaded Convolutional Network; Xception, Extreme Inception Convolutional Network; RF, Random Forest; K-ELM, Kernel Extreme Learning Machine; S-RCNN-WSCR, Spatial R-CNN with Weighted Soft Classification Regularization; MEOWDL-ER, Multi-Encoder Optimized Weighted DL for ER; FME, Facial-Micro Expression; VAR, Video Action Recognition; MDT-GCN, Multi-Domain Transfer Graph Convolutional Network; STM, Short-Term Memory module; HSF, High-level Semantic Features; GNM, Graph Neural Model; LR, Logistic Regression; SE, Squeeze-and-Excitation module; HAR, Human Activity Recognition; TCN, Temporal Convolutional Network; CBAM, Convolutional Block Attention Module; MER, Multimodal Emotion Recognition; Viola-Jones, Viola-Jones cascade object/face detector; EfficientNet, Efficient scalable Convolutional Network (compound scaling); VGG16, Visual Geometry Group 16-layer network; HGSO-DLFER, Henry Gas Solubility Optimization with Deep Learning-based Facial Emotion Recognition; SRCN-DFER, Sequential Recurrent Convolutional Network for Dynamic FER; Wav2Vec2.0, Wav2Vec 2.0 self-supervised speech representation model; ConvLSTM, Convolutional Long Short-Term Memory network; STResNet, Spatio-Temporal ResNet; CBAM-ResNet34, ResNet-34 backbone augmented with a Convolutional Block Attention Module; SMILE-VLM, Self-supervised Multi-view representation LEarning Vision-Language Model; kNN, k-Nearest Neighbors; GNN, Graph Neural Network; MCA/TCA, Multi-/Temporal-Channel Attention; HuBERT, Hidden-unit BERT (self-supervised speech representation model); Swin Transformer, Shifted Window Transformer; ELM-RBF, Extreme Learning Machine with Radial Basis Function kernel; BERT, Bidirectional Encoder Representations from Transformers; BiLSTM, Bidirectional Long Short-Term Memory network; RBF, Radial Basis Function; KAN, Kolmogorov-Arnold Network; SSL, Self-Supervised Learning; cGAN, Conditional GAN; SENet, Squeeze-and-Excitation Network; GRU, Gated Recurrent Unit. Subjects Column: H, Healthy People / P, Patients / N, No Participants.

ROBIS was developed to assess bias in the review process itself rather than to appraise the methodological quality of individual primary studies. Because the included studies were predominantly algorithmic validations conducted on pre-existing benchmark datasets rather than clinical trials or observational studies, standard study-level appraisal tools (e.g., Cochrane RoB 2, MMAT) were not directly applicable either. To complement the review-level ROBIS assessment, we therefore constructed a study-level quality indicator, informed by, but not equivalent to, the ROBIS domains, adapted to the specific context of dataset-driven deep-learning studies. Three domains were considered: dataset eligibility and representativeness, data collection and annotation quality, and reporting/synthesis of results. Signalling questions for each domain (Supplementary Table SA1) were answered per study, and each of the 60 included studies was assigned an overall Low or Mid quality-concern category (Supplementary Table SA2). No reporting/publication bias assessment (e.g., funnel plot analysis) was conducted, as such methods are designed for quantitative meta-analyses and are not applicable to the narrative/thematic synthesis performed here.

## 3 Results

### 3.1 Overview

To provide a structured overview of the current state of research, the results of our review are presented through two complementary tables. Table 3 offers a compact comparative summary of the articles on automatic emotion recognition, revealing clear patterns across application domains, sensing modalities, algorithmic choices, and evaluation practices. In particular, the table highlights the predominance of vision-based approaches (29 out of 60 reviewed contributions), followed by speech/audio-based methods (15/60). From a modeling perspective, deep convolutional architectures are the most widely adopted (at least 29 contributions), frequently combined with recurrent or attention-based components in hybrid pipelines. It also exposes a marked variability in reported accuracy values, which is closely linked to differences in task formulation, number of emotion classes, and data availability, as well as a notable imbalance between methodological sophistication and real-world technological deployment. As a secondary outcome of the analysis of the results of the Tables 1, 3, a compilation of emotion-related datasets used in this research field is presented in Tables 5, 6. These tables also includes additional datasets found in related studies to the ones in this literature search.

**TABLE 5:** Facial emotion recognition and multimodal databases.

Database	Type				Quantity	Subjects	Access
	I	V	A	EEG			
(1) CK+	X				920	123	R
(2) Caltech faces	X				450	27	P
(3) CMU MultiPIE	X				750,000	337	R+F
(4) NIST	X				3,248	1,573	R
(5) Kaggle CK+48	X				5,000	-	P
(6) KDEF	X				4,900	70	R
(7) AFEW		X			54 movies	-	A
(8) TFD	X				112,234	-	R
(9) IEMOCAP		X			302	-	R
(10) Kaggle FER 2013	X				35,887	-	P
(11) SAVEE			X		480	-	P
(12) IIM indore	X				1,302	186	R
(13) AffectNet	X				400,000	-	A
(14) AM-FED		X			242	242 users	R
(15) Emotionet	X				1.000.000	-	A
(16) RECOLA		X	X		9 h 30 m	-	R
(17) VAM		X			947	-	R
(18) MELD		X	X		13,000	-	R
(19) DFEPN		X			1,897	-	R
(20) UNBC-McMaster		X			200	-	R
(21) WSEFEP	X				2,940	-	R
(22) Oulu-CASIA		X			2,880	80	R
(23) MMI		X			2,900	75	R
(24) RAF-DB	X				15,339	-	R
(25) RAVDESS	X				7,356	24	P
(26) SFEW 2.0	X				1,766	-	R
(27) JAFFE	X				213	10	R
(28) ADFES		X			1,344	22	R
(29) RaFD	X				8,040	67	R
(30) ExpW	X				91,793	-	A
(31) IMFDB	X				34,512	100	R
(32) BioVid		X			700 + trials	87	R
(33) SpaExp		X			720 sequences	54	R
(34) CAFE	X				1,192	-	R
(35) EMO-DB			X		535 utterances	10	R
(36) EMOVO			X		588 utterances	6	R
(37) SFEW 1.0	X				≈ 700	-	R
(38) DEAP				X	40 trials each	32	R
(39) MAHNOB-HCI		X			20 sessions	27	R
(40) SEED		X			-	15	R
(41) SEED-IV				X	-	15	R
(42) FACES	X				2052	171	R
(43) NVIE		X			2,200 vid seq	215	R
(44) GRE2019	X				1,275	-	R
(45) SMIC		X			328 seq	20	R
(46) CAER-S	X				70,000+	-	R
(47) RML		X			720	-	R
(48) POFA	X				110	-	R+F
(49) JACFEE	X				56	-	R+F
(50) NimStim	X				646	43	R
(51) 4DME		X			Hundreds	10s	R
(52) RFFd	X				≈ 2K	-	R
(53) AVEC2021		X	X		≈ 7.5 h	-	R
(54) FERG*	X				55769	6	P

Type = I: Image, V: Video, A: Audio / Access = R: Research purposes, P: Publicly available, R+F: Research + fees, A: Access request/restricted.

**TABLE 6:** Facial emotion recognition and multimodal databases.

Database	Type				Quantity	Subjects	Access
	I	V	A	EEG			
(55) DISFA		X			≈ 130K frames	27	R
(56) DISFA +		X			≈ 484K	27	R
(57) RaF	X				8,040	67	R
(58) NTUST-IRL	X				640x6	20	R
(59) CelebA	X				202599	10177	R
(60) MOSEI		X	X		223453 segments	1,000	R
(61) CREMA-D		X	X		7,442 clips	91	R
(62) Kinematics-400/700		X			400 clips/650K clips	A lot	R
(63) MiT		X			> 1,000,000 videos	A lot	R
(64) UCF-101		X			13,320 videos	-	R
(65) HMDB-SV		X			6,766	-	R
(66) Diving48		X			≈ 18,000 clips	-	R
(67) BU-3DFE		X			≈ 2,500 models in 3D	≈ 100	R
(68) BU-4DFE		X			Hundreds of seq	≈ 100	R
(69) Bosphorus	X				4,666 scans	105	R
(70) Spontaneous		X			-	≈ 40	R
(71) SEWA		X	X		-	-	R
(72) SEED-V		X		X	-	≈ 16	R
(73) TESS			X		2,800 clips	2	R
(74) Emotion-gait		X			2,177 seq	-	R
(75) Emotion-walk		X			-	-	R
(76) FER+	X				≈ 35K	-	R
(77) EMOTIC	X				≈ 18–23 K	-	R
(78) BP4D	X	X			2.6 TB	41	R
(79) MEVIEW		X			41 videos	16	R
(80) M-LFW-F	X				≈ 9–10k	5 749	R
(81) DFEW		X			16,372	-	R
(82) CASME II		X			3 000 seq	26	R
(83) SAMM		X			159 clips	32	R
(84) DREAMER				X	18 clips	23	R
(85) MPED				X	28 videos	23	R
(86) Oulu-CASIA	X	X			400 seq	80	R
(87) MERTI-apps		X		X	3, 236 records	62	R
(88) Google SC v0.02			X		105,000 clips	2,618	P
(89) POFA	X				110	60	R
(90) MAFW	X	X	X		10.045 images	-	R
(91) ESVG		X	X		455 videos	16	R
(92) Aff-Wild2		X	X		548 videos	-	R
(93) Emilya dataset		X	X		8,206 samples	11	R
(94) BRED		X	X		215 seq	30	R
(95) eNTERFACE05		X	X		1,260 videos	42	R
(96) Twitter I	X				≈ 5,000 images	-	R
(97) Twitter II	X				≈ 4,000–6,000 images	-	R
(98) Abstract	X				280 images	-	R
(99) ArtPhoto	X				≈ 800 images	-	R
(100) MVSA-single	X				4,800 images	-	R
(101) MVSA-multiple*	X				4,800 images	-	R
(102) CASME		X			≈ 255–300 seq	35	R
(103) FERV39K		X			38.935 clips	-	R
(104) CMU-MOSI		X			2.199 seg	93	R
(105) CH-SIMS		X	X		≈ 2000–2,500 seg	60	R
(106) MER-MULTI		X	X		≈ 6,000–7,000 samples	-	R
(107) FERPlus	X				35.887 images	-	R
(108) PRISM(SZ,AD)				X	40–60 min per individual	170	R

Type = I: Image, V: Video, A: Audio / Access = R: Research purposes, P: Publicly available, R+F: Research + fees, A: Access request/restricted.

Aggregating dataset usage across the 60 studies of the final PRISMA-based corpus (Table 3) reveals a markedly concentrated reuse pattern. CK+ is the single most frequently used benchmark (11/60 studies, 18.6%), followed by IEMOCAP (10/60, 16.7%) and DEAP (8/60, 13.3%); the five most-cited datasets (CK+, IEMOCAP, DEAP, RAVDESS, and RAF-DB) together account for 35.9% of all dataset citations recorded in the corpus. Conversely, 31 of the 50 distinct datasets identified in this corpus are cited in only a single study, indicating a pronounced long-tail distribution. Notably, the two most-reused benchmarks, CK+ and IEMOCAP, were originally released in 2010 and 2008, respectively, suggesting that evaluation practice in this field continues to rely disproportionately on a small set of legacy corpora despite the more recent availability of larger and more demographically diverse datasets (e.g., AffectNet, RAF-DB).

In terms of sensing strategy, unimodal approaches (43/60, 71.6%) considerably outnumber multimodal ones (17/60, 28.3%) across the corpus. Multimodal designs are most common when EEG or physiological signals are involved (e.g., EEG combined with facial expressions or eye movement) and in wearable-computing contexts combining inertial and optomyographic sensors, whereas the majority of purely vision-based (FER) and purely speech-based (SER) studies remain single-modality despite the fusion techniques increasingly available in the field.

Participant involvement follows a similarly skewed pattern: the majority of studies (36/60, 60.0%) report no human participants at all, relying exclusively on pre-existing benchmark datasets; 23/60 (38.3%) involve healthy volunteers recruited specifically for the study; and only 1/60 (1.7%) involves a clinical patient population Li D et al. (2022), discussed in Section 3.2.3). This distribution underscores a broader pattern already noted in this review: algorithmic validation on existing corpora considerably outweighs primary data collection with real users, and validation involving clinical populations is nearly absent from the field as currently practiced.

In terms of temporal distribution, the corpus spans publications from 2019 to 2025, with a clear concentration in the most recent years: 39.0% of studies were published in 2023–2024 alone, and a further 22.0% in 2025, together accounting for 61% of the reviewed literature. This recency reflects the field’s rapid ongoing growth rather than a mature, settled body of work, and is consistent with the architectural trend noted throughout this review toward lightweight and attention-based models, which have only become computationally practical in the last few years.

Finally, with respect to real-time deployment rates, only 10 of the 60 studies in the corpus (16.7%) report any form of hardware-level or real-time validation, such as inference latency, frame rate on embedded devices, or live deployment on a physical platform: Handrich et al. (2021), Hwang et al. (2022), Pu and Nie (2023), Subramanian et al. (2022), Kang and Kim (2022), Li et al. (2024), Mai et al. (2025), Sohail et al. (2024), Sun and Cai (2024), and Radoi and Cioroiu (2024) (full details available in Table 3). The remaining 83.3% report exclusively offline classification accuracy on held-out benchmark splits. Among the 9 studies reporting such validation, evidence points to a consistent gap between benchmark accuracy and real-world readiness: Li et al. (2024) sustains real-time inference on a Raspberry Pi 4B with only 0.82M parameters, Pu and Nie (2023) deploys a pruned facial expression model (approximately 41% parameter reduction) on a NAO robot in a live human-computer interaction experiment, achieving recognition probabilities above 0.7 for most emotions but only 0.61 for disgust, and Kang and Kim (2022) reports over 80% reduction in computation time for a lightweight physiological-signal model without substantial accuracy loss. This scarcity of hardware-level validation present in roughly one in seven studies indicates that computational efficiency and deployment readiness remain a secondary concern relative to offline accuracy maximization in the majority of the reviewed literature.

As Table 3 illustrates, deep convolutional architectures dominate the corpus (at least 30 of the 60 contributions), frequently combined with recurrent or attention-based components; readers seeking the specific algorithmic choice, dataset, and reported accuracy for any individual study can consult this table directly, rather than the narrative synthesis in this section repeating this level of detail.

Together, the four presented tables enable the identification of dominant research trends, methodological gaps, and persistent challenges that are discussed in the following subsections.

### 3.2 Categorization by application domain

The following subsections present a categorization of the main application areas stemming from these emotion recognition methods, focusing on four primary use cases or deployment contexts reported by each study: i) human-computer interaction, ii) human-robot interaction, iii) healthcare, rehabilitation, and assistive technologies, and iv) specialized scenarios: traffic safety and wearable computing. This classification was performed based on the primary use case or deployment context reported by each study. Studies addressing purely algorithmic validation without a stated deployment context were grouped separately under “Algorithmic validation and benchmarking (no specific application)”.

Across the 60 studies reviewed, automatic emotion recognition emerges as a rapidly evolving research area that integrates computer vision, speech processing, and neurophysiological sensing. In quantitative terms, visual-based approaches dominate the literature, accounting for >50% of the reviewed works, with a strong emphasis on facial emotion recognition (FER). Speech-based emotion recognition (SER) represents the second most explored modality, addressed in approximately >25% of the studies, while physiological and neurophysiological approaches, mainly based on EEG signals, appear in a smaller subset of works, typically within experimental or laboratory-oriented settings. In parallel, a growing number of studies explore multimodal combinations of vision, speech, text, and physiological signals, reflecting an increasing interest in richer and more robust affective representations (Shahzad et al., 2023; Dikbiyik et al., 2025; Güler and Akbulut, 2025).

#### 3.2.1 Human-computer interaction (HCI)

HCI constitutes the most recurrent application area (19 studies), where emotion recognition is incorporated to adapt system behavior, personalize feedback, or enhance user experience. Across the reviewed studies, two convergent trends emerge regardless of modality (facial, speech, or multimodal): a shift from handcrafted features toward end-to-end CNN pipelines, and a subsequent shift from raw CNN backbones toward lightweight or attention-augmented variants intended for real-time or embedded deployment. However, closer comparison across studies reveals that this architectural convergence is not matched by a convergence in evaluation rigor, and several studies that report near-identical pipelines differ mainly in which pretrained backbone or metaheuristic they substitute, rather than in the underlying mechanism being tested.

A recurrent pattern in this subset is the pairing of a pretrained CNN feature extractor with a metaheuristic algorithm for hyperparameter tuning and an autoencoder-based classifier. Two studies exemplify this pattern almost exactly: one couples MobileNet with Henry Gas Solubility Optimization and a Nadam-trained autoencoder (AlEisa et al., 2023), while the other couples GoogleNet with a modified Earthworm Optimization algorithm and a quantum autoencoder (Alrowais et al., 2023). Both are evaluated on the same CK + subset (920 images, 8 classes) under the same 70:30 and 80:20 splits, and both report accuracies above 98%, with gains attributed to the choice of metaheuristic rather than to any change in how facial emotion is represented. Neither study evaluates on a second, independently collected dataset, so it remains unclear whether the reported gains reflect genuine feature-extraction improvements or dataset-specific overfitting to a single small corpus. This pattern illustrates a broader limitation of the HCI subset: architectural novelty is frequently confined to the optimizer or the classifier head, while the evaluation protocol (single corpus, single split, comparison only against other pretrained CNNs) remains constant across papers, limiting the strength of any claimed improvement.

Reproducibility and generalization are rarely addressed directly. Among the eight HCI-related studies examined in depth, only one performs an explicit cross-corpus evaluation, training on one facial-expression dataset and testing on another (Dar et al., 2022). The results are informative precisely because they contradict the near-perfect accuracies reported within single corpora: the same EfficientNet-based model that reaches 95%–100% accuracy on CK + or FERG drops to 30%–58% when trained on one dataset (e.g., FER-2013) and tested on another (e.g., CK+), a gap the authors attribute to differences in illumination, camera distance, and the demographic and cultural composition of each dataset. This cross-corpus result is a rare direct measurement of generalization in the reviewed literature, and its absence from the remaining studies is a methodological gap rather than a minor omission: without it, accuracy figures above 95%–98% reported in single-corpus evaluations (Jaffar et al., 2024; Guo et al., 2023; AlEisa et al., 2023; Alrowais et al., 2023) cannot be assumed to transfer to unconstrained, real-world HCI settings. None of the eight studies reports public availability of code or trained models, which further constrains independent verification of the reported figures.

Deployment readiness is addressed unevenly. Most studies report only offline classification accuracy on held-out test splits, without measuring inference latency, memory footprint, or behavior under an actual human-computer interaction loop. Two studies are exceptions and are informative for contrast. First, a lightweight speech emotion recognition model is benchmarked not only for accuracy but for inference time across three hardware tiers, including a Raspberry Pi 4B, where it sustains more than 10 inferences within a 3-s speech segment despite a parameter count of only 0.82M (Li et al., 2024); this is one of the few studies in the subset that treats computational cost as a first-class evaluation criterion rather than a secondary remark.

Second, a facial expression recognition system is not only pruned for a smaller memory footprint (approximately 41% reduction) but is actually deployed on a NAO robot and tested in a live interaction experiment with human participants (Pu and Nie, 2023); the resulting recognition probabilities in this deployed setting (0.61 for disgust versus above 0.7 for most other emotions) are visibly lower than the accuracies reported on static benchmark datasets, and the authors attribute this gap to the limited number of training samples for underrepresented emotion classes. This contrast is instructive: the two studies that go beyond benchmark accuracy to test real hardware or live interaction are also the only two that report a measurable accuracy drop between benchmark and deployment conditions, suggesting that offline accuracy in this literature is a systematically optimistic proxy for HCI readiness.

In the demographic scope are additional limitations that are often acknowledged by the authors themselves but rarely addressed methodologically. The personality-assessment system built around a NAO robot and the Big Five model, for instance, is validated on only 16 participants (Jaffar et al., 2024), and the authors explicitly note that the presence of a humanoid robot may itself alter participants’ non-verbal behavior (a Hawthorne-type effect), which is a validity concern specific to HCI settings that is absent from purely algorithmic FER or SER benchmarks. Similarly, several of the facial-expression datasets reused across studies in this subset (e.g., CK+, JAFFE) are demographically narrow. CK+ is predominantly composed of female and African-American subjects in a controlled lab setting, and JAFFE consists exclusively of Japanese female subjects, a constraint that propagates into every downstream HCI study that reuses these corpora without supplementing them with more diverse data.

Taken together, these studies indicate that HCI-oriented emotion recognition has matured architecturally (lightweight backbones, attention modules, and metaheuristic tuning are now standard) but has not matured methodologically at the same pace: cross-corpus generalization, code/data availability, and live-deployment testing remain the exception rather than the norm, and the few studies that do test these dimensions report meaningfully lower performance than the single-corpus accuracies that dominate the field’s reported results.

#### 3.2.2 Human-robot interaction (HRI)

HRI constitutes another recurrent application area comprising 8 studies in our selection, where emotion recognition and behavioral analysis are integrated into social or collaborative robotic systems to foster more natural, adaptive, and intuitive interactions. Within this framework, research explores distinct conceptual paradigms to decode human affect. On one hand, physical expression platforms heavily rely on surface non-verbal channels.

For instance, a notable baseline involves the extraction of multimodal non-verbal cues such as facial expressions via CNN coupled with head and body pose estimations to analyze live user personality traits and affective responses during dynamic interactions with humanoid robots like NAO Jaffar et al. (2024). In a similar vein, advanced architectures utilize Sequential Recurrent Convolution Networks (SRCN), combining CNN extracted feature vectors with Long Short-Term Memory (LSTM) blocks to synergistically process dynamic facial emotions and wireless speech commands during collaborative tasks Hwang et al. (2022). This temporal tracking is crucial, as it facilitates continuous emotion monitoring over long-term interactions, thereby allowing for robust behavioral modeling and the dynamic adaptation of the robot’s operational logic Kansizoglou et al. (2022). Furthermore, the integration of metaheuristic optimization algorithms, such as the modified Earthworm Optimization Algorithm (EWA) coupled with deep learning classifiers, has proven highly effective in refining visual feature selection and boosting classification accuracy within human-computer interfaces designed for robotic environments Alrowais et al. (2023).

Conversely, an alternative methodological branch argues that surface features like voice or facial expressions are governed by the somatic nervous system and can be intentionally manipulated or masked by the user. To capture authentic, “inner” emotional states, researchers have shifted toward autonomic nervous system (ANS) physiological signals, implementing lightweight 1D Convolutional Autoencoders optimized via knowledge distillation to classify Photoplethysmogram (PPG) and Galvanic Skin Response (GSR) data under the tight hardware and real-time processing constraints of robotic platforms Kang and Kim (2022).

Architectural advances within this category are heavily driven by the technical limitations of real-world environments. Specifically, deep learning models frequently struggle with severe intra-class variations caused by shifts in user identity or changing environmental illumination. To mitigate this issue, frameworks incorporating Conditional Generative Adversarial Networks (cGAN) have been proposed to explicitly disentangle facial expressions from identity factors, utilizing cycle consistency losses to ensure structural stability in HRI applications Deng et al. (2019). Furthermore, to bypass the classic issues of visual occlusion that occur from a fixed scene or robot-centric perspective, alternative perception paradigms explore egocentric (first-person) perspectives via eyewear cameras, utilizing CNN-LSTM setups to track head and eye gestures in real time Marina-Miranda and Traver (2022).

The studies reviewed in this subsection Deng et al. (2019); Kang and Kim (2022); Hwang et al. (2022) primarily address domain-agnostic, fundamental algorithmic principles centered on bidirectional communication, hardware optimization, and general behavioral loops, rather than clinically-oriented rehabilitation. This subsection is therefore kept independent of Section 3.2.3, to draw a clear line between general social/collaborative interaction mechanisms and the highly specialized, clinically-oriented frameworks designed for physical rehabilitation and healthcare assistance.

#### 3.2.3 Healthcare, rehabilitation, and assistive technologies

Healthcare, rehabilitation, and assistive applications account for 10 studies. Efforts have primarily focused on the objective quantification of mental states to reduce reliance on subjective self-reports in diagnosing conditions like depression and stress (Othmani et al., 2021; Subramanian et al., 2022).

Physiological and neurophysiological approaches, particularly EEG-based systems, have advanced markedly. Recent studies employ graph neural networks (GNN), transformers, autoencoders, and ensemble CNN–LSTM architectures to capture spatial temporal dynamics of brain activity (Bagherzadeh et al., 2024; Mai et al., 2025; Ghous et al., 2025). Optimization strategies grounded in swarm intelligence and graph-based learning further enhance channel selection (Yildirim et al., 2021; Gilakjani and Al Osman, 2023).

Despite this motivating framing, direct evidence connecting emotion recognition to rehabilitation-specific outcomes remains scarce within the reviewed corpus. Only Li D et al. (2022) involves a clinical patient population in this subsection, fusing EEG topographic maps with facial expressions via a CBAM_ResNet34 classifier to recognize four emotional states in individuals with hearing impairment (78.32% accuracy for the fused modality, versus 69.43% for EEG alone and 67.90% for facial expressions alone). However, the reported outcome is diagnostic classification accuracy, not a rehabilitation-relevant metric such as therapy adherence, motor-learning gain, or engagement duration, and the study does not report any subsequent intervention informed by the recognized emotional state. Subramanian et al. (2022) proposes a decision-support framework in which a real-time emotion recognition system is paired with a digital twin, allowing a physician to simulate a treatment before applying it. While framed for personalized healthcare, this is a clinician-mediated recommendation tool rather than an autonomous or robotic closed loop, and it is validated only on healthy volunteers (3 participants) rather than patients. Across the 10 studies reviewed in this subsection, none implements a closed control loop in which recognized affect automatically modulates a rehabilitation-relevant parameter (e.g., task difficulty, robotic assistance level, feedback pacing), and no study reports a clinical validation trial linking emotion-aware feedback to therapy adherence or motor-learning outcomes.

#### 3.2.4 Specialized scenarios: traffic safety and wearable computing

Traffic safety is addressed in 6 studies, including driver-state and anger detection systems (Ding et al., 2024). Wearable computing (2 studies) is exemplified by emotion-aware smart glasses (Marinova et al., 2025). Efficient FER approaches are especially suitable for these scenarios, where latency, energy consumption, and portability constitute key deployment constraints (Pu and Nie, 2023; Alrowais et al., 2023).

Multimodal emotion recognition represents the most prominent trend in these scenarios. Early feature-level concatenation has been replaced by hierarchical attention, graph alignment, and neuro-inspired fusion models. Systems combining facial expressions, speech, and physiological signals consistently outperform unimodal counterparts, often by margins of 15–25 percentage points (Thant and Panitanarak, 2025; Goncalves et al., 2025; Güler and Akbulut, 2025).

#### 3.2.5 Algorithmic validation and benchmarking (no specific application)

Fourteen of the fifty-nine studies (23.7%) are restricted to algorithmic validation under controlled conditions, without a clearly defined deployment context. Beyond the catalog of architectures, the dominant pattern in this subset is a pronounced disconnect between intra-dataset and cross-dataset (cross-corpus) performance, which is rarely reported and, when it is, reveals a substantial drop in accuracy.

The clearest case is Saurav et al. (2021), which combines a classical texture descriptor (DLTP) with PCA and a Kernel-ELM classifier, achieving very high intra-dataset cross-validation figures (CK+: 99.76%; RaF: 99.72%; JAFFE: 96.71%; KDEF: 93.98%; RAF-DB: 78.75%) that collapse under cross-dataset validation (CK+ → JAFFE: 42.25%; RaF → JAFFE: 41.31%). Choi and Song (2020) reports an equivalent pattern in micro-expression recognition: 71.34% (SMIC) and 73.98% (CASME II) intra-dataset versus only 58.54%–63.57% in SMICCASME II cross-validation. Morales-Vargas et al. (2019) confirms the same pattern with a methodologically distinct approach, an interpretable fuzzy model based on Action Units rather than deep learning: training on CK+ with Active Appearance Models as the landmark extractor yields 90.8 ± 14% intra-dataset, but evaluating that same model cross-dataset on RaFD drops accuracy to 69 ± 1%. The consistency of this gap across such heterogeneous architectures (ELM, implicit temporal descriptors, fuzzy inference systems) suggests the problem is not specific to a particular model family but rather to the dominant evaluation protocol in this subset of studies.

Morales-Vargas et al. (2019) further surfaces two findings rarely made explicit elsewhere in the reviewed literature. First, a reproducibility issue tied to the landmark extractor, independent of the classifier: using the same fuzzy model on CK+, accuracy shifts from 90.8% (AAM-based landmarks) to only 76% (Deva-Ramanan); on RaFD, from 81% (Deva-Ramanan) to just 45% (OpenFace). In other words, the choice of landmark extractor, a preprocessing component usually treated as trivial, can produce accuracy swings of up to 36 percentage points with the same downstream classifier, which severely complicates cross-study comparability when this factor goes unreported or uncontrolled. Second, the study quantifies the cost of removing the dependency on a reference “neutral” frame (needed to normalize landmark displacements): replacing the true neutral state with a synthetic one derived from the dataset mean lowers accuracy from 90.8% to 70% on CK+ and from 81% to 61% on RaFD, a degradation that exposes a convenience assumption (availability of a neutral reference frame) rarely met in real deployment scenarios, and one the study itself acknowledges as a practical limitation.

A second pattern is that architectural gains attributed to novel mechanisms are frequently confounded with the effect of the underlying backbone. In Su et al. (2021), switching the backbone from VGG16 to DenseNet raises accuracy from 76.92% to 88.63% on Oulu-CASIA almost 12 points while the proposed refinement mechanism (RHPN over HPN) contributes only an additional +1.74 points. Most of the gain attributed to the “proposed method” is, in fact, an effect of the pretrained feature extractor.

Third, studies differ in whether they address readiness for real-world deployment. Vo et al. (2020) identifies that VGG16 degrades under input image size variation (a realistic condition in RAF-DB/AffectNet) and compensates with a pyramid architecture incorporating super-resolution (88.98% RAF-DB; 89.75% FER+; 60.68%–63.77% AffectNet), but does not report the computational cost of the additional super-resolution branches. Choi and Song (2020) does report this: its compact variant reduces parameters to 8% of the original model (718K vs. 9.19M) with only marginal accuracy degradation, though its “in-the-wild” validation (MAHNOB-HCI, MEVIEW) is purely qualitative (t-SNE), not quantitative. Kansizoglou et al. (2022) represents a distinct variant of the same underlying issue: rather than categorical classification, it targets continuous arousal/valence estimation via facial landmarks and an LSTM architecture, validated under a reasonably strict leave-one-speakers-group-out protocol (RECOLA; CCC of 0.446 for arousal and 0.676 for valence, competitive with the state of the art). However, the study performs no cross-corpus validation whatsoever, it is confined to a single dataset, and acknowledges a structural, rather than architectural, limitation: estimation error for arousal (MSE0.039) is consistently 4–5 times higher than for valence (MSE0.009) across all test subjects, a pattern the authors attribute to arousal being inherently harder to capture from static facial geometry alone, requiring complementary audio input, a modality limitation, not an architecture limitation, that is rarely discussed this explicitly in the purely categorical studies elsewhere in this section. Finally, a metric-selection problem that can mask real model weaknesses is evident. Kapaliya et al. (2024) reports 89% weighted accuracy but an F1-score of only 0.42 and an AUC of 0.734, indicating class imbalance left unresolved by overall accuracy. Pham et al. (2019), by contrast, proposes not a new architecture but a re-classification layer based on Action-Unit image retrieval (drawing on EmotioNet, 1M images) applied on top of existing networks, yielding modest gains (+1.0–1.2 pp) confined to the subset of predictions flagged as “unreliable”, an approach oriented toward mitigating errors in an already-deployed system rather than maximizing headline accuracy, but one not validated beyond FER2013.

Taken together, of the eight studies analyzed in depth so far, none reports publicly available code, and only three (Saurav et al., 2021; Choi and Song, 2020; Morales-Vargas et al., 2019) provide any figure enabling assessment of computational cost, model size, or sensitivity to preprocessing components. Moreover, five of the eight studies that do report cross-dataset or cross-corpus evaluation show drops of between 15 and 48 percentage points relative to their intra-dataset figure which, taken as a whole, directly calls into question the representativeness of the state-of-the-art comparison tables that dominate this literature, nearly all of which are built on intra-dataset figures.

## 4 Discussion

Although emotional models have advanced considerably, the integration of rule-based approaches with emotional models to produce contextually relevant behaviors remains underexplored (Heredia et al., 2022; Li Y. et al., 2022). Affective computing, supported by advanced in DL-based emotion recognition, offers a promising approach to address this limitation (Chen and Luo, 2023). Such, technologies can decode emotional signals more effectively and deliver adaptive actions aligned with user states (Ghayoumi and Pourebadi, 2019).

### 4.1 Methodological advancements and dataset challenges

As detailed in Tables 5, 6 the reused benchmark datasets vary substantially in size, modality, and demographic scope (e.g., CK+ with 920 images and AffectNet with 400,000). This differences contribute to, in our interpretation, the generalization gaps discussed throughout this section.

Despite the potential of integration-based models, our performed analysis of the reviewed literature points to persistent methodological challenges that limit the generalizability of the current findings in assistive contexts. Real-time facial emotion recognition methods exhibit substantial variability in performance across demographic groups. Models trained on limited or non-representative datasets can systematially underperform for users from underrepresented age groups, ethnicities, or cultural backgrounds. Within the 10 studies analyzed in clinical and assistive applications (Section 3.2.3), this limitation is particularly critical. Although recent works have advanced markedly in physiological computing using EEG-based architectures such GNNs and Transformers (Bagherzadeh et al., 2024; Mai et al., 2025; Ghous et al., 2025), they remain heavily dependent on laboratory-controlled datasets.

### 4.2 Opportunities and limitations of emotion recognition in HRI

Emotion recognition in HRI has evolved from simple state detection into a key requirement for long-term human-robot collaboration (Leite et al., 2013). Recent work also shows that deep learning models are now designed to handle real-world challenges, such as environmental noise and user-identity variations (Deng et al., 2019), as evidenced by the findings in Section 3.2.2.

However, based on the reviewed evidence, a major limitation remains the tension between data accessibility and emotional authenticity. Current data modalities present a clear trade-off. External channels such as facial expressions and speech are non-invasive and rich in detail, but users can easily fake or mask them due to social pressure and cultural display rules (Ekman, 1993). In contrast, internal physiological signals (such as PPG and GSR) offer an authentic, unmanipulated indication of emotional states because they are regulated by the autonomic nervous system and are largely resistant to voluntary control (Picard, 2000). Yet, capturing these signals requires uncomfortable wearable sensors or heavy data compression to meet the strict real-time hardware constraints of mobile robots. Similarly, while egocentric cameras in eyewear reduce visual occlusions, they raise concerns about user comfort and social acceptance.

### 4.3 Technical trade-offs: efficiency and interpretability of HRI systems

From a methodological standpoint, the field has shifted from single-modality pattern recognition toward multimodal and attention-driven architectures (Dikbiyik et al., 2025; Wang et al., 2023). While these architectures mirror emotive appraisal processes more closely, computational efficiency remains a challenge for real-time robotics. Fusion methods entail trade-offs between accuracy and latency, highlighting the need for lightweight algorithms that balance speed and performance for mobile and assistive robotic hardware (Er, 2020; Li et al., 2024). Unlike desktop applications, social robots often rely on embedded systems in which CPU/GPU resources must be shared between emotion recognition, simultaneous localization and mapping (SLAM), and motor control. To mitigate this computational bottleneck, three main architectural alternatives are reported in the robotics literature. First, edge-cloud hybrid computing allows platforms to offload resource-intensive affective deep-learning models to remote cloud infrastructure while retaining safety-critical control tasks strictly onboard (Kehoe et al., 2015). Second, dedicated low-power hardware accelerators, such as NVIDIA Jetson modules or edge tensor processing units (TPUs), can execute complex emotion recognition networks on dedicated hardware. This apporach reduces demands on the main CPU resources shared by navigation loops (Le et al., 2026). Finally, model optimization techniques such as post-training quantization and knowledge distillationcan compress neural networks, enabling lightweight affective models to run efficiently alongside real-time control loops on low-specification hardware (Han et al., 2015).

To address the “black-box” nature of these models, interpretability has emerged as an important consideration in the responsible development of HRI. Strategies such as fuzzy logic reasoning (Morales-Vargas et al., 2019) and action unit semantic modeling (Shahzad et al., 2023) enhance transparency while maintaining predictive power.

### 4.4 Applications, ethics, and user engagement

Previous research on affective human–robot interaction suggests that user engagement and emotional motivation significantly influence the usability and acceptance of technologies (Guerrero et al., 2013; Abbasi et al., 2024). Moreover, the ability of a a robot’s ability to display “empathy” by adjusting its behavior to the user’s affective state of the user, an instance of what we define in this manuscript as emotional intelligence, can directly impact the social agency attributed to the machine. This behavior represents an instance of what we define in this manuscript as emotional intelligence. Such perceptions of the robot have beena proposed as adeterminant of long-term therapeutic adherence (Feil-Seifer and Matarić, 2011).

However, the rapidly evolving regulatory and ethical landscape increasingly constrains this engagement-driven design goal. Recent systematic reviews of the literature on the ethics of emotion recognition technologies highlight persistent concerns about algorithmic bias, the sensitivity of emotional data, and the risk of harm in consequential settings such as healthcare (Katirai, 2023). Additionally, the EU AI Act (2024) generally prohibits the deployment of emotion detection systems, permitting them only under a narrowly interpreted “medical exception” for strictly therapeutic applications (Vecellio Segate, 2024). This regulatory framing is directly relevant to the assistive and rehabilitation-oriented systems discussed in this review (Section 3.2.3). Specifically it implies that emotion-aware behavioral adjustments in robotic assistance would need to be explicitly framed, validated, and documented as therapeutic to remain lawful within the EU. Within the reviewed corpus, however, this mechanism is only indirectly supported: Subramanian et al. (2022) link recognized affect to personalized healthcare decision-support but validates the system only on healthy volunteers and does not measure engagement or adherence as an outcome. As noted in Section 3.2.3, no study in the corpus evaluates whether emotion-aware behavioral adjustments improves engagement, motivation, or therapeutic adherence in practice.

### 4.5 Future perspectives and recommendations

When cross-dataset evaluation or real-world deployment is attempted, such as tracking patient engagement or diagnosing depression (Sun and Cai, 2024; Subramanian et al., 2022), the lack of demographic diversity in training data may reduce classification performance and limit demographic generalizability. For technologies aimed at tailored recovery protocols (Li D. et al., 2022), such underperformance could prevent the robotic control loop from detecting the emotional distress or frustration of older adults or underrepresented groups.

In our view, future research should move beyond static analysis to continuous temporal modeling. This approach would allow robots to build behavioral profiles of users over time and evolve from passive tools into adaptive collaborators (Dautenhahn, 2007). We identify three main research gaps that should guide future research in this field. The first is the isolation of multimodal approaches: despite their theoretical benefits, few lightweight models efficiently fuse external cues with internal physiological data.

The second is the perception-action mismatch: in our interpretation, models can effectively decode affective and psychophysiological states such as stress or anger, but lack the decision-making rules needed to translate that perception into safe and appropriate physical responses. The findings of this review support this observation (Section 3.2.3). Of the 10 studies addressing healthcare and assistive applications, only one (Li D. et al., 2022) involves a clinical patient population, one reports a rehabilitation-relevant outcome measure (e.g., therapy adherence or motor-learning gain) or a closed loop linking recognized affect to an adaptive robotic or therapeutic action. This finding echoes a broader concern raised in the affective-HRI literature about the scarcity of studies that evaluate how specific robotic actions driven by recognized emotions affect therapy or interaction outcomes (Cañamero, 2005).

Finally, the third gap concers contextual grounding. Systems often assume that an emotion means the same thing in any situation. Future work should therefore develop context-aware architectures in which the interpretation generated by the robot is dynamically moderated by the ongoing task.

Reinforcement learning offers opportunities for personalization, because RL-based systems can adjust responses in real time based on user affect. However, their trial-and-error nature, in our assessment, introduces inherent risks during early training phases. As a research perspective rather than a conclusion derived from the reviewed studies, we recommend that assistive scenarios prioritize constraint-aware, hybrid RL architectures that ensure adaptability does not compromise user wellbeing or physical safety during interaction. We therefore recommend prioritizing constraint-aware, hybrid RL architectures in assistive scenarios. This should provide adaptability without compromising user wellbeing or physical safety during interaction.

Emotion-aware engagement, could, in principle, optimize performance by maintaining a balanced challenge–skill dynamic. This approach could, transform recognition from a diagnostic tool into an active regulator of HRI. Most HRI studies are conducted under controlled conditions with small, homogeneous samples, making it difficult to assess long-term efficacy and the socio-psychological impact of sustained robotic assistance. For example, Abbasi et al. (2024) included only 41 participants. While laboratory performance is high (Pham et al., 2019; Sajjad and Kwon, 2020), robustness under real-world variations in lighting, noise, and cultural expression remains insufficiently tested. This may be relevant in social robotics, where the robot is perceived not just as a tool but as a social agent. We consider empirical demonstration of this transition, rather than its assumption, an open task for the field.

The collection of sensitive physiological data also introduces ethical and legal challenges, especially when robots operate in private or domestic spaces. Data protection frameworks must evolve alongside algorithmic sophistication to guarantee confidentiality and prevent “emotional hacking” or manipulation, and inclusive design principles are vital for ensuring equitable deployment. In our view, performance metrics alone are insufficient. Psychological safety, user trust, and emotional authenticity should be considered equally fundamental indicators of robotic acceptance within the socio-psychological framework of assistive technology.

Future research should prioritize longitudinal evaluation and ecological realism. A primary challenge in this transition is the current lack of closed-loop integration. Although systems detect states effectively, approaches that autonomously adapt robotic assistance or therapy intensity in real time remain exceptionally rare (Hwang et al., 2022; Kansizoglou et al., 2022; Li D. et al., 2022; Sun and Cai, 2024).

Onboard hardware limitations also restrict the implementation of continuous closed-loop interventions in unconstrained environments. Transitioning from passive state estimation to an active control loop will require processing complex affective models without compromising real-time execution or draining shared computational resources.As a research perspective rather than a conclusion drawn from the reviewed evidence, we recommend that future work in general human-computer interfaces (HCI) focus on deploying streamlined MobileNet-style or SwishNet backbones (Dar et al., 2022; Behzad, 2025) capable of operating within strict and energy consumption thresholds (Pu and Nie, 2023). For mobile and collaborative robotics, this involves overcoming the shared CPU/GPU bottleneck caused by the simultaneous execution of navigation (SLAM) and motor control. Addressing this bottleneck requires dedicated edge-hardware accelerators (Le et al., 2026) combined with deeply compressed architectures like 1D Convolutional Autoencoders for physiological streaming (Kang and Kim, 2022). We therefore consider, as a forward-looking research direction rather than a finding of this review, that the future of closed-loop affective computing relies on co-designing hardware-software solutions. Advanced pruning and quantization (Han et al., 2015) could help prevent computational overhead from introducing safety-critical latencies into the operational logic of the system.

In our assessment, the field is moving toward a new paradigm that values interpretability and human alignment alongside quantitative accuracy. The transition from laboratory-based classification to real-world affective human-robot collaboration will be defined by the ability of robots to align their actions with the evolving psychological and physical states of the users.

## 5 Conclusion

Beyond methodological advances, emotion recognition technologies are attracting rapidly growing research interest in the evolution of HRI and assistive robotics. In these domains, affect detection is increasingly viewed as a critical enabling component for socially assistive robots, empathetic rehabilitation platforms, and personalized healthcare systems. However, while the theoretical benefits of this integration are widely acknowledged, this review highlights that technical maturity in facial and multimodal recognition has not yet translated into widespread, real-time application. A significant gap persists in closing the control loop between affect detection and robotic response. Consequently, the practical efficacy and positive user outcomes traditionally attributed to emotional awareness remain largely unverified in closed-loop systems.

Deploying such systems outside laboratory conditions introduces critical challenges related to real-time onboard processing, physical safety, and socio-psychological alignment in unconstrained, real-world environments. To validate the assumed benefits of affective HRI and ensure long-term usability, future developments must transition from offline algorithmic validation toward evaluating “affect-driven” control architectures *in vivo*. Much of the evidence synthesized in this review originates from Human-Computer Interaction and generic emotion recognition methods not involving physical robotics applications. This underscores the need for future work to actively transfer and validate these methods within embodied, robotic platforms. Ultimately, the integration of emotional intelligence into assistive robots represents a necessary shift toward systems that adapt to the complexity of human experience, offering a promising but still developing pathway toward a safer, more effective, and more equitable collaboration between humans and robots.

## Data Availability

The original contributions presented in the study are included in the article/Supplementary Material, further inquiries can be directed to the corresponding author.
