# Supplementary material for: Emotion recognition and human-robot interaction: a systematic review of methods and applications
Source: Front Robot AI. 2026 Sep 11;13:1891746. doi: 10.3389/frobt.2026.1891746 (PMC13612989; doi:10.3389/frobt.2026.1891746)
Supplement: Supplementary file 1 [file Supplementaryfile1.pdf]

A APPENDIX: RISK AND BIAS

Supplementary Table 1. Signalling questions used to appraise study-level quality concerns, informed by ROBIS domains and adapted to dataset-driven emotion-recognition studies.

**Domain 1: Dataset eligibility and representativeness**
1. Is the dataset used publicly available or clearly documented?
2. Is the dataset size adequate relative to the number of emotion classes considered?
3. Is the demographic composition of the dataset (age, gender, ethnicity) reported or discussed?

**Domain 2: Data collection and annotation quality**
4. Is the data collection or acquisition protocol clearly described?
5. Are ground-truth emotion labels validated (e.g., by independent annotators or established coding schemes)?
6. Are potential confounding factors (illumination, occlusion, recording conditions, cultural display rules) acknowledged?

**Domain 3: Reporting and synthesis of results**
7. Is model performance evaluated with more than a single aggregate metric (e.g., F1-score, AUC, not only accuracy)?
8. Is cross-dataset or cross-corpus validation reported?
9. Are code, trained models, or sufficient implementation detail available for independent verification?

Note: These questions are not part of the original ROBIS instrument, which assesses risk of bias at the level of the systematic review process rather than of individual primary studies (see Table **??**). They were instead adapted from the underlying ROBIS logic to the specific context of dataset-driven deep-learning studies. Each question was answered Yes/No/Unclear per study. Studies with predominantly "Yes" answers across all three domains were classified as *Low* quality-concern; studies with one or more "No"/"Unclear" answers, particularly in Domain 1.

Supplementary Table 2. Study-level quality-concern category for the included studies (informed by ROBIS-derived domains)

Authors	Risk of Bias
Nawaz et al. (2025)	Low
Kansizoglou et al. (2022)	Low
Dar et al. (2022)	Low
Pham et al. (2019)	Low
Borgalli and Surve (2022)	Low
Kapaliya et al. (2024)	Low
Saurav et al. (2021)	Low
Su et al. (2021)	Low
Alharbawee and Pugeault (2024)	Low
Avani et al. (2021)	Low
AlEisa et al. (2023)	Low
Hwang et al. (2022)	Low
Alrowais et al. (2023)	Low
Dong et al. (2023)	Low
Vo et al. (2020)	Low
Luna-Jiménez et al. (2021)	Low
Marinova et al. (2025)	Mid
Subramanian et al. (2022)	Mid
Morales-Vargas et al. (2019)	Low
Li et al. (2022a)	Mid
Colaco and Han (2025)	Low
Behzad (2025)	Low
Li et al. (2022b)	Low
Choi and Song (2020)	Low
Kim et al. (2021)	Low
Kang and Kim (2022)	Low
Yildirim et al. (2021)	Low
Gilakjani and Al Osman (2023)	Low
Li et al. (2024)	Low
Cao et al. (2025)	Low
Guo et al. (2023)	Low
Ding et al. (2024)	Mid
Marimpis et al. (2020)	Low
Er (2020)	Low
Ghous et al. (2025)	Low
Dikbiyik et al. (2025)	Low
Sajjad et al. (2020)	Low
Bagherzadeh et al. (2024)	Low
Mai et al. (2025)	Low
Thant and Panitanarak (2025)	Low
Deeb et al. (2025)	Low
Priyadarshani et al. (2024)	Mid
Chen et al. (2024)	Mid
Ho et al. (2020)	Low
Shahzad et al. (2023)	Low
Wang et al. (2023)	Low
Wu et al. (2020)	Mid
Dal Rí et al. (2023)	Low
Li et al. (2025)	Low
Deng et al. (2019)	Low
Pu and Nie (2023)	Low
Sohail et al. (2024)	Mid
Lai et al. (2020)	Low
Marina-Miranda and Traver (2022)	Mid
Goncalves et al. (2025)	Low
Sun and Cai (2024)	Mid
Jaffar et al. (2024)	Mid
Radoi and Cioroiu (2024)	Low
Güler and Akbulut (2025)	Low

REFERENCES

AlEisa, H. N., Alrowais, F., Negm, N., Almalki, N., Khalid, M., Marzouk, R., et al. (2023). Henry gas solubility optimization with deep learning based facial emotion recognition for human computer interface. *IEEE Access* 11, 62233–62241. doi:10.1109/ACCESS.2023.3284457

Alharbawee, L. and Pugeault, N. (2024). Generative adversarial networks for facial expression recognition in the wild. *International Journal of Computing and Digital Systems* 16, 1259–1282. doi:10.12785/ijcds/160193

Alrowais, F., Negm, N., Khalid, M., Almalki, N., Marzouk, R., Mohamed, A., et al. (2023). Modified earthworm optimization with deep learning assisted emotion recognition for human computer interface. *Ieee Access* 11, 35089–35096. doi:10.1007/s11571-020-09638-x

Avani, V. S., Shaila, S., and Vadivel, A. (2021). Geometrical features of lips using the properties of parabola for recognizing facial expression. *Cognitive Neurodynamics* 15, 481–499. doi:10.1007/s11571-020-09638-x

Bagherzadeh, S., Shalbaf, A., Shoeibi, A., Jafari, M., Tan, R.-S., and Acharya, U. R. (2024). Developing an eeg-based emotion recognition using ensemble deep learning methods and fusion of brain effective connectivity maps. *IEEe Access* 12, 50949–50965. doi:10.1109/ACCESS.2024.3384303

Behzad, M. (2025). Smile-vlm: Self-supervised multi-view representation learning using vision-language model for 3d/4d facial expression recognition. *IEEE Access* 13, 143831–143842. doi:10.1109/ACCESS.2025.3598019

Borgalli, R. A. and Surve, S. (2022). Facial emotion recognition through detection of facial action units and their intensity. *Scientific Visualization* 14. doi:10.1109/10.26583/sv.14.1.06

Cao, R., Wang, Y., Wu, X., Jin, S., and Niu, H. (2025). A lightweight tri-stream feature fusion network for speech emotion recognition. *IEEE Access* doi:10.1109/ACCESS.2025.3587607

Chen, J., Tu, P., Qiu, Z., Zheng, Z., and Chen, Z. (2024). Intelligent human-computer interaction dialog model based on end to end neural networks and emotion information. *IEEE Access* 12, 102880–102892. doi:10.1109/ACCESS.2024.3432860

Choi, D. Y. and Song, B. C. (2020). Facial micro-expression recognition using two-dimensional landmark feature maps. *IEEE Access* 8, 121549–121563. doi:10.1109/ACCESS.2020.3006958

Colaco, S. J. and Han, D. S. (2025). Scalable context-based facial emotion recognition using facial landmarks and attention mechanism. *IEEE Access* doi:10.1109/ACCESS.2025.3534328

Dal Rí, F. A., Ciardi, F. C., and Conci, N. (2023). Speech emotion recognition and deep learning: an extensive validation using convolutional neural networks. *IEEE Access* 11, 116638–116649. doi:10.1109/ACCESS.2023.3326071

Dar, T., Javed, A., Bourouis, S., Hussein, H. S., and Alshazly, H. (2022). Efficient-swishnet based system for facial emotion recognition. *IEEE Access* 10, 71311–71328. doi:10.1109/ACCESS.2022.3188730

Deeb, B. M., Savchenko, A. V., and Makarov, I. (2025). Enhancing emotion recognition in speech based on self-supervised learning: Cross-attention fusion of acoustic and semantic features. *IEEE Access* doi:10.1109/ACCESS.2025.3554454

Deng, J., Pang, G., Zhang, Z., Pang, Z., Yang, H., and Yang, G. (2019). cgan based facial expression recognition for human-robot interaction. *IEEE Access* 7, 9848–9859. doi:10.1109/10.1109/ACCESS.2019.2891668

Dikbiyik, E., Demir, O., and Dogan, B. (2025). Bimer: design and implementation of a bimodal emotion recognition system enhanced by data augmentation techniques. *IEEE Access* doi:10.1109/ACCESS.2025.3559339

Ding, T., Zhang, K., Gao, S., Miao, X., and Xi, J. (2024). A multimodal driver anger recognition method based on context-awareness. *IEEE Access* 12, 118533–118550. doi:10.1109/ACCESS.2024.3422383

Dong, Z., Li, X., Lin, B., and Xie, F. (2023). Multi-net fusion: Exploring a brain-inspired neural network model for facial expression recognition. *IEEE Access* 11, 93804–93814. doi:10.1109/ACCESS.2023.3310398

Er, M. B. (2020). A novel approach for classification of speech emotions based on deep and acoustic features. *IEEE Access* 8, 221640–221653. doi:10.1109/ACCESS.2020.3043201

Ghous, G., Najam, S., Alshehri, M., Alshahrani, A., AIQahtani, Y., Jalal, A., et al. (2025). Attention-driven emotion recognition in eeg: A transformer-based approach with cross-dataset fine-tuning. *IEEE Access* doi:10.1109/ACCESS.2025.3561137

Gilakjani, S. S. and Al Osman, H. (2023). A graph neural network for eeg-based emotion recognition with contrastive learning and generative adversarial neural network data augmentation. *IEEE Access* 12, 113–130. doi:10.1109/ACCESS.2023.3344476

Goncalves, L., Chou, H.-C., Salman, A. N., Lee, C.-C., and Busso, C. (2025). Jointly learning from unimodal and multimodal-rated labels in audio-visual emotion recognition. *IEEE Open Journal of Signal Processing* doi:10.1109/OJSP.2025.3530274

Güler, S. E. and Akbulut, F. P. (2025). Multimodal emotion recognition: Emotion classification through the integration of eeg and facial expressions. *IEEE Access* doi:10.1109/ACCESS.2025.3538642

Guo, Y., Zhou, Y., Xiong, X., Jiang, X., Tian, H., and Zhang, Q. (2023). A multi-feature fusion speech emotion recognition method based on frequency band division and improved residual network. *IEEE Access* 11, 86013–86024. doi:10.1109/ACCESS.2023.3299822

Ho, N.-H., Yang, H.-J., Kim, S.-H., and Lee, G. (2020). Multimodal approach of speech emotion recognition using multi-level multi-head fusion attention-based recurrent neural network. *IEEE Access* 8, 61672–61686. doi:10.1109/ACCESS.2020.2984368

Hwang, C.-L., Deng, Y.-C., and Pu, S.-E. (2022). Human–robot collaboration using sequential-recurrent-convolution-network-based dynamic face emotion and wireless speech command recognitions. *Ieee Access* 11, 37269–37282. doi:10.1109/ACCESS.2022.3228825

Jaffar, A., Ali, S., Iqbal, K. F., Ayaz, Y., Ansari, A. R., Fayyaz, M. A., et al. (2024). A comprehensive multimodal humanoid system for personality assessment based on the big five model. *IEEE Access* 12, 84261–84272. doi:10.1109/ACCESS.2024.3412931

Kang, D.-H. and Kim, D.-H. (2022). 1d convolutional autoencoder-based ppg and gsr signals for real-time emotion classification. *IEEe Access* 10, 91332–91345. doi:10.1109/ACCESS.2022.3201342

Kansizoglou, I., Misirlis, E., Tsintotas, K., and Gasteratos, A. (2022). Continuous emotion recognition for long-term behavior modeling through recurrent neural networks. *Technologies* 10, 59. doi:10.3390/technologies10030059

Kapaliya, S., Swain, D., Sharma, R., Varyani, K., and Thakar, J. (2024). Facial emotion recognition with deep neural network: A study of visual geometry group-16 (vgg16) technique with data augmentation for improved precision. *Pertanika Journal of Science & Technology* 32. doi:10.47836/pjst.32.5.02

Kim, D. H., Anvarov, F., Lee, J. M., and Song, B. C. (2021). Metric-based attention feature learning for video action recognition. *IEEE access* 9, 39218–39228. doi:10.1109/ACCESS.2021.3064934

Lai, H., Chen, H., and Wu, S. (2020). Different contextual window sizes based rnns for multimodal emotion detection in interactive conversations. *IEEE Access* 8, 119516–119526. doi:10.1109/ACCESS.2020.3005664

Li, D., Liu, J., Yang, Y., Hou, F., Song, H., Song, Y., et al. (2022a). Emotion recognition of subjects with hearing impairment based on fusion of facial expression and eeg topographic map. *IEEE Transactions on Neural Systems and Rehabilitation Engineering* 31, 437–445. doi:10.1109/TNSRE.2022.3225948

Li, H., Zhao, D., Wang, J., and Wang, D. (2024). A lightweight multi-scale model for speech emotion recognition. *IEEE Access* doi:10.1109/ACCESS.2024.3432813

Li, J., Dai, X., Yan, R., Tang, C., and Li, Y. (2025). Multi-anchor adaptive fusion and bi-focus attention for enhanced gait-based emotion recognition. *Scientific Reports* 15, 14946. doi:10.1038/s41598-025-97922-3

Li, X., Cheng, S., Li, Y., Behzad, M., Shen, J., Zafeiriou, S., et al. (2022b). 4dme: A spontaneous 4d micro-expression dataset with multimodalities. *IEEE Transactions on Affective Computing* 14, 3031–3047. doi:10.1109/TAFFC.2022.3182342

Luna-Jiménez, C., Kleinlein, R., Griol, D., Callejas, Z., Montero, J. M., and Fernández-Martínez, F. (2021). A proposal for multimodal emotion recognition using aural transformers and action units on ravdess dataset. *Applied Sciences* 12, 327. doi:10.3390/app12010327

Mai, T. D. T., Vu, D.-Q., Phuong, H. N., and Phung, T.-N. (2025). Eeg-skdnet: A self-knowledge distillation model with scaled weights for emotion recognition from eeg signals. *IEEE Access* doi:10.1109/ACCESS.2025.3594671

Marimpis, A. D., Dimitriadis, S. I., and Goebel, R. (2020). A multiplex connectivity map of valence-arousal emotional model. *Ieee Access* 8, 170928–170938. doi:10.1109/ACCESS.2020.3025370

Marina-Miranda, J. and Traver, V. J. (2022). Head and eye egocentric gesture recognition for human-robot interaction using eyewear cameras. *IEEE Robotics and Automation Letters* 7, 7067–7074. doi:10.1109/LRA.2022.3180442

Marinova, M., Chona, E., Kotevski, A., Sazdov, B., Kiprijanovska, I., Stankoski, S., et al. (2025). Deep learning for facial expression and human activity recognition using smart glasses. *IEEE Access* doi:10.1109/ACCESS.2025.3551610

Morales-Vargas, E., Reyes-García, C., and Peregrina-Barreto, H. (2019). On the use of action units and fuzzy explanatory models for facial expression recognition. *PloS one* 14, e0223563. doi:10.1371/journal.pone.0223563

Nawaz, U., Saeed, Z., and Atif, K. (2025). A novel transformer-based approach for adult's facial emotion recognition. *IEEE Access* doi:10.1109/ACCESS.2025.3555510

Pham, T. T. D., Kim, S., Lu, Y., Jung, S.-W., and Won, C.-S. (2019). Facial action units-based image retrieval for facial expression recognition. *IEEE Access* 7, 5200–5207. doi:10.1109/ACCESS.2018.2889852

Priyadarshani, M., Kumar, P., Babulal, K. S., Rajput, D. S., and Patel, H. (2024). Human brain waves study using eeg and deep learning for emotion recognition. *IEEE Access* doi:10.1109/ACCESS.2024.3427822

Pu, J. and Nie, X. (2023). Convolutional channel attentional facial expression recognition network and its application in human–computer interaction. *IEEE Access* 11, 129412–129424. doi:10.1109/ACCESS.2023.3333381

Radoi, A. and Cioroiu, G. (2024). Uncertainty-based learning of a lightweight model for multimodal emotion recognition. *IEEE Access* doi:10.1109/ACCESS.2024.3450674

Sajjad, M., Kwon, S., et al. (2020). Clustering-based speech emotion recognition by incorporating learned features and deep bilstm. *IEEE access* 8, 79861–79875. doi:10.1109/ACCESS.2020.2990405

Saurav, S., Saini, R., and Singh, S. (2021). Facial expression recognition using dynamic local ternary patterns with kernel extreme learning machine classifier. *IEEE Access* 9, 120844–120868. doi:10.1109/ACCESS.2021.3108029

Shahzad, H., Bhatti, S. M., Jaffar, A., Rashid, M., and Akram, S. (2023). Multi-modal cnn features fusion for emotion recognition: A modified xception model. *IEEE Access* 11, 94281–94289. doi:10.1109/ACCESS.2023.3310428

Sohail, M., Shoukat, I. A., Fatima, H., Jafri, M. R., Yaqub, M. A., Liotta, A., et al. (2024). Deep learning based multi pose human face matching system. *IEEE Access* 12, 26046–26061. doi:10.1109/ACCESS.2024.3366451

Su, W., Zhang, H., Su, Y., and Yu, J. (2021). Facial expression recognition with confidence guided refined horizontal pyramid network. *IEEE Access* 9, 50321–50331. doi:10.1109/ACCESS.2021.3069468

Subramanian, B., Kim, J., Maray, M., and Paul, A. (2022). Digital Twin Model: A Real-Time Emotion Recognition System for Personalized Healthcare. *IEEE Access* 10, 81155–81165. doi:10.1109/ACCESS.2022.3193941

Sun, X. and Cai, Z. (2024). Research on an eye control method based on the fusion of facial expression and gaze intention recognition. *Applied Sciences* 14, 10520. doi:10.3390/app142210520

Thant, A. M. and Panitanarak, T. (2025). Emotion recognition through advanced signal fusion and kolmogorov-arnold networks. *IEEE Access* doi:10.1109/ACCESS.2025.3572583

Vo, T.-H., Lee, G.-S., Yang, H.-J., and Kim, S.-H. (2020). Pyramid with super resolution for in-the-wild facial expression recognition. *IEEE Access* 8, 131988–132001. doi:10.1109/ACCESS.2020.3010018

Wang, S., Qu, J., Zhang, Y., and Zhang, Y. (2023). Multimodal emotion recognition from eeg signals and facial expressions. *IEEE Access* 11, 33061–33068. doi:10.1109/ACCESS.2023.3263670

Wu, D., Zhang, J., and Zhao, Q. (2020). Multimodal fused emotion recognition about expression-eeg interaction and collaboration using deep learning. *IEEE access* 8, 133180–133189. doi:10.1109/ACCESS.2020.3010311

Yildirim, E., Kaya, Y., and Kiliç, F. (2021). A channel selection method for emotion recognition from eeg based on swarm-intelligence algorithms. *IEEE Access* 9, 109889–109902. doi:10.1109/ACCESS.2021.3100638
